# Novel mutations of the *SRF* gene in Chinese sporadic conotruncal heart defect patients

**DOI:** 10.1186/s12881-020-01032-y

**Published:** 2020-05-07

**Authors:** Xu Mengmeng, Xu Yuejuan, Chen Sun, Lu Yanan, Li Fen, Sun Kun

**Affiliations:** 1 Department of Pediatric Cardiology, Xinhua Hospital, Shanghai Jiao Tong University School of Medicine, No.1665 Kongjiang road, Shanghai, 200092 China; 2 Shanghai Children’s Medical Center, Shanghai Jiao Tong University School of Medicine, No. 1678, Dongfang Road, Shanghai, 200127 China

**Keywords:** SRF, Conotruncal heart defects, Mutation

## Abstract

**Background:**

Conotruncal heart defects (CTDs) are a group of congenital heart malformations that cause anomalies of cardiac outflow tracts. In the past few decades, many genes related to CTDs have been reported. Serum response factor (SRF) is a ubiquitous nuclear protein that acts as transcription factor, and SRF was found to be a critical factor in heart development and to be strongly expressed in the myocardium of the developing mouse and chicken hearts. The targeted inactivation of SRF during heart development leads to embryonic lethality and myocardial defects in mice.

**Methods:**

To illustrate the relationship between SRF and human heart defects, we screened *SRF* mutations in 527 CTD patients, a cross sectional study. DNA was extracted from peripheral leukocyte cells for target sequencing. The mutations of SRF were detected and validated by Sanger sequencing. The affection of the mutations on wild-type protein was analyzed by in silico softwares. Western blot and real time PCR were used to analyze the changes of the expression of the mutant mRNA and protein. In addition, we carried out dual luciferase reporter assay to explore the transcriptional activity of the mutant SRF.

**Results:**

Among the target sequencing results of 527 patients, two novel mutations (Mut1: c.821A > G p.G274D, the adenine(A) was mutated to guanine(G) at position 821 of the SRF gene coding sequences (CDS), lead to the Glycine(G) mutated to Asparticacid(D) at position 274 of the SRF protein amino acid sequences; Mut2: c.880G > T p.G294C, the guanine(G) was mutated to thymine (T) at position 880 of the SRF CDS, lead to the Glycine(G) mutated to Cysteine (C) at position 294 of the SRF protein amino acid sequences.) of *SRF* (NM_003131.4) were identified. Western blotting and real-time PCR showed that there were no obvious differences between the protein expression and mRNA transcription of mutants and wild-type SRF. A dual luciferase reporter assay showed that both SRF mutants (G274D and G294C) impaired SRF transcriptional activity at the *SRF* promoter and atrial natriuretic factor (*ANF*) promoter (*p* < 0.05), additionally, the mutants displayed reduced synergism with GATA4.

**Conclusion:**

These results suggest that SRF-p.G274D and SRF-p.G294C may have potential pathogenic effects.

## Background

Congenital heart disease (CHD) is the most common congenital malformation in live births (0.5–1%), and is a major cause of high mortality in newborns and children [1]. Conotruncal heart defects (CTDs) mainly include tetralogy of Fallot (TOF), double outlet of right ventricle (DORV), transposition of the great arteries (TGA), pulmonary atresia/ventricle septal defect (PA/VSD), and persistent truncus arteriosus (PTA), and account for approximately 20% ~ 30% of CHD [1, 2]. A large number of studies have found that genetic factors are the major causes of CTDs [3–7]. To date, nucleotide mutations in more than 20 genes and chromosomal abnormalities have been demonstrated to be involved in syndromic and nonsyndromic CTDs. The 22q11.2 microdeletion syndrome and the 8p23.1 duplication syndrome tend to be associated with different types of CTDs [8–11]. Duplications of 1q21.1 have been implicated in the pathogenesis of TOF [12]. Transcription factors and cofactors, such as GATA4, NKx2.5, GATA6 and TBX1, are involved in CTD pathogenesis, as demonstrated by numerous research reports [4, 11]. Mutations in the JAG1-NOTCH signaling pathway have also been detected in patients with isolated TOF or Alagille syndrome [13].

Serum response factor (SRF) is a member of the MADS-box (MCM1, Agamous, Deficiens, SRF) transcription factor family. SRF is widely expressed and binds to the conserved CC(A/T)^6^GG DNA sequences (CArG box), which is mainly located in the promoter regions of muscle and growth factor genes [14–21]. SRF binds to the CArG-box and acts as an anchoring protein that binds to other factors and efficiently regulates target gene transcription. SRF is one of the key transcription factors during cardiac development and participates in the regulation of cardiac-related gene expression in its dimeric form [16, 19, 20]. The development of the heart is a complex process with multi-stage that requires the regulation and coordination of various genes. As previously reported, SRF is essential for normal heart development and maturation because it influences the function of myocardin which regulates BMP10, a member of the gene regulatory network of the heart [22–24]. Moreover, the conditional mutagenesis of murine SRF leads to a slow heartbeat and a poorly developed interventricular groove and ventricular wall [25]. These reports suggest that the mutation of SRF may result in the dysregulation of cardiac development.

Our study aimed to screen mutations in the SRF gene in a cohort consisting of 527 patients diagnosed with CTDs and to test the functional influence of the identified SRF mutants. We identified two novel missense mutations in *SRF* in the patients’ cohort. Luciferase assay results suggested that the two mutations impair SRF function and might be implicated in the pathogenesis of CTDs in Chinese patients.

## Methods

### Patients and samples

A total of 527 sporadic nonsyndromic CTD patients were recruited from XinHua Hospital and Shanghai Children’s Medical Center [26] (Table 1). CHD was diagnosed by echocardiography, cardiac catheterization, or surgery. The exclusion criteria included the following: 1) chromosome karyotype confirmed as trisomy 21; 2) family history of CHD; 3) 22q11 microdeletion/duplication with cardiac malformation. Three hundred healthy individuals were also recruited as controls. This study was approved by the Medical Ethics Committee of Shanghai Children’s Medical Center and Xinhua Hospital. All parents were informed of the purpose and significance of the experiment and signed an informed consent form. The mean age and sex ratio were matched between CTD patients and the control group.

**Table 1:** Diagnoses of the study objects

Diagnoses	Numbers
Pulmonary atresia/ventricular septal defect	97
Tetralogy of Fallot	220
Double outlet right ventricle	98
Transposition of the great arteries	90
Truncus arteriosus	9
Interrupted aortic arch	13
Total	527

Peripheral blood was collected, and genomic DNA was extracted using a QIAamp DNA Blood Mini Kit (QIAGEN, Hilden, Germany) according to the manufacturer’s instructions. A UV spectrophotometer (NanoDrop Technologies, Wilmington, DE, USA) was used to analyze the DNA purity. All DNA samples were stored at − 80 °C for future use.

### Targeted sequencing

DNA samples were sent to TianHao Biotechnology Co, Ltd., China (a commercial provider) to screen for SRF mutations. Primers were designed by using Primer3 software, and the PCR products were labeled with a barcode for subsequent detection. Base incorporation was performed according to the manufacturer’s standard measurement protocol, and cluster generation was performed on a MiSeq Benchtop sequencer (Illumina, lnc, San Diego, CA, USA) [27].

### Mutation validation

The mutations detected by targeted sequencing were validated by Sanger sequencing. The primers (SRF-seq) were designed by using Primer5 for the *SRF* sequence (NC_000006.12) (Table 2). PCR amplification was performed, and PCR products were sequenced using an ABI 3730 sequencer (Applied Biosystems, Foster City, CA, USA).

**Table 2:** Primer pairs used for the experimental methods

Primers	Forward (5′ → 3′)	Reverse (5′ → 3′)
**SRF-seq**	TGCCAGGTAGTGTTTTCTAAGTG	GGCCCCTATTCACCTTCCTT
**Mut-G274D**	CACCAACCTGCCGGATACAACCTCCACCA	TGGTGGAGGTTGTATCCGGCAGGTTGGTG
**Mut-G294C**	GCAAGTCAGCAGCTGCCCCTCCTTTCC	GGAAAGGAGGGGCAGCTGCTGACTTGC
**RT-SRF**	ACTCTCCACCCCGTTCAGAC	TGGTGCACTTGAATGGCCTG
**GAPDH**	GAGTCAACGGATTTGGTCGT	TGATTTTGGAGGGATCTCG

### Multiple SRF sequence alignments and online function prediction

To evaluate the conservation of mutated sites in the SRF protein, the protein sequences of human (NP_003122.1), *Macaca mulatta* (XP_001093365), chimpanzee (XP_518487), mouse (NP_065239.1), rat (NP_001102772), dog (XP_852302.1), bovine (NP_001192945), chicken (NP_001239070), *xenopus tropicalis* (XP_002942523), and zebrafish (NP_001103996) were obtained from and the Protein database of National Center of Biotechnology Information (NCBI: https://www.ncbi.nlm.nih.gov/home/protein/) and aligned with ClustalX. The impact of the mutation on SRF proteins was also predicted by MutationTaster (http://www.mutationtaster.org/), SIFT (http://sift.jcvc.org/www/SIFT_enst_submit.html) and Polyphen-2 (http://genetics.bwh.havard.edu/pph2/).

### Plasmids construction

The SRF open reading frame (ORF) clone was purchased from OriGene Technologies (Catalog No: SC118177). Primers for site-directed mutagenesis (Mut-G274D forward/reverse and Mut-G294C: forward/reverse) were designed online (https://www.genomics.agilent.com/primerDesignProgram.jsp) (Table 3), and PCR amplification products were digested using DpnI (NEB, Catalog: R0176S) before being transfected into DH5α and cultured on ampicillin dishes at 37 °C for 14 h. Selected successful mutant colonies were grown in 120 ml LB medium (Beyotime, ST158), and then plasmids were extracted from the bacteria solution.

**Table 3:** Function prediction of the SRF mutants (SRF: NP_003122.1)

Patient No.	Diagnosis	Mutations	Mutation Taster	SIFT	Polyphen-2
**P070**	PA/VSD	SRF.pG274D	Disease-causing	0.02	1.0/0.946
**P124**	TOF/RAA	SRF.pG294C	Disease-causing	0.02	0.999/0.936

**Note. 1** A SIFT score < 0.05 means damage, and a Polyphen-2 score > 0.85 (HumDiv/HumVar) means damage, according to the descriptions of the two web-based tools

To construct the SRF*luc* reporter plasmid, the genomic sequence of *SRF* (NC_000006.12) was obtained from the Gene database of the Gene databases of National Center of Biotechnology Information (NCBI: https://www.ncbi.nlm.nih.gov/gene/). A fragment beginning approximately 1.2 kb upstream of the transcription initiation site of *SRF* was amplified (sense-primer: 5′ –C G G G G T A C C T T T C T G C T G G G C A C G G T G G T - 3′, antisense-primer: 5′ - A T G G C G A G G C C G C T C C T T A T A A G C T T G G G- 3′) from the DNA of a healthy individual and was cloned into the pGL3-basic vector (Promega, USA) between the KpnI and HindIII sites. The atrial natriuretic factor (ANF) promoter plasmid was a kind gift from Professor Mona Nemer [28]. The full-length cDNAs for GATA4 expression constructs in the pcDNA3.1(+) vector were previously generated in our laboratory [29]. All plasmid DNA was confirmed and Sanger sequenced by the Beijing Genomics Institute (BGI) in China.

### Cell culture and transfection

All cell lines used in this study were purchased from the cell bank of the Chinese Academy of Sciences. The HEK293 (Catalog No. GNHu 1) and NIH3T3 (Catalog No. GNM 6) cell lines were cultured in medium containing Dulbecco’s Modified Eagle’s Media (DMEM), 10% fetal bovine serum (FBS), penicillin (100 unit/ml), and streptomycin (100 μg/ml) before transfection. Cells were transfected with Fugene HD (Promega, E2311) according to the manufacturer’s protocol. Transfected NIH3T3 cells were starved by culturing in DMEM supplemented with 0.5% FBS for 48 h.

### Western bloting and real-time PCR

HEK293 cells were transfected with wild-type or mutant SRF plasmid in 12-well plates 24 h after plating, and were harvested after 48 h transfection. Cells were washed with cold DPBS and then lysed on ice using RIPA lysis buffer with PMSF (1/100) for 30 min for total protein extraction. For RNA extraction, the transfected cells were lysed with TRIzol for 10 min.

For Western blotting, 25 μg of protein per sample was separated using 8% SDS-PAGE (Beyotime, China) and transferred onto PVDF membranes (0.45 μm, Life Technology, ThermoFisher, Scientific). The membranes were blocked for 2 h using 5% skim milk in Tris-buffered saline with 0.2% Tween-20. A rabbit anti-human SRF antibody (1:2000 in 5% BSA, Genview, USA) or mouse anti-human actin antibody (1:2000 in 5% BSA, Genview, USA) was used to incubate the membranes at 4 °C overnight. The next day, the membranes were incubated with a horseradish peroxidase-conjugated goat anti-rabbit antibody or goat anti-mouse antibody (secondary antibodies) for 2 h. Immobilon ECL (Millipore, USA) and a ChemiDoc XRS+ system (Bio-Rad, USA) were used to visualize the protein bands.

For qPCR, 1000 ng of total RNA was reverse transcribed to synthesize cDNA using a PrimeScript**™**RT reagent kit (Takara, RR037A). Primers (RT-SRF) for the SRF sequence (NM_003131.4, obtained from the Gene database of NCBI) (forward: 5′-A C T C T C C A C C C C G T T C A G A C-3′/reverse: 5′-T G G T G C A C T T G A A T G G C C T G-3′) and the reference GAPDH sequence (forward: 5′-G A G T C A A C G G A T T T G G T C G T-3′/reverse: 5′-T G A T T T T G G A G G G A T C T C G-3′) were designed using Primer5 (Table 3). Comparative Ct (^**ΔΔ**^Ct) relative quantitation analysis was used to analyze the Ct value in qPCR, and experiments were repeated three times.

### Luciferase assay

To assess the activation of mutant SRF on the ANF promoter alone, we cotransfected 200 ng of wild-type/mutant SRF constructs, and 66.67 ng of ANF-reporter plasmids; and when in synergy with GATA4, we cotransfected 100 ng of wild-type/mutant SRF constructs, 100 ng of empty vector or 100 ng GATA4 construct, and 66.67 ng of ANF-reporter plasmids per well into starved cells (24 h after plating, the 10% FBS medium was replaced with 0.5% FBS medium before transfection) of 48-well plates. In addition, we cotransfected mutant or wild-type constructs or the pcDNA3.1(+) vector and the SRF-reporter plasmid into NIH3T3 cells using Fugene HD (Promega, USA), and pRL-TK (Promega) was used as an internal control reporter plasmid.

We compared the ANF promoter and SRF promoter expression in the different transfection groups when NIH3T3 cells were cultured in 10% FBS medium or 0.5% FBS medium 48 h after transfection. The luciferase activity of cells was determined by using a Dual-Glo luciferase assay system (Promega, E2920).

### Statistical analysis

The data were analysed by SPSS 23.0 for Windows. All results are presented as the mean ± SEM. Mean of two continuous normally distributed variables were compared by independent samples Student’s test. A value of *p* < 0.05 was considered significant.

## Results

### Two missense mutations were identified in SRF in CTD patients

**Fig. 1:**
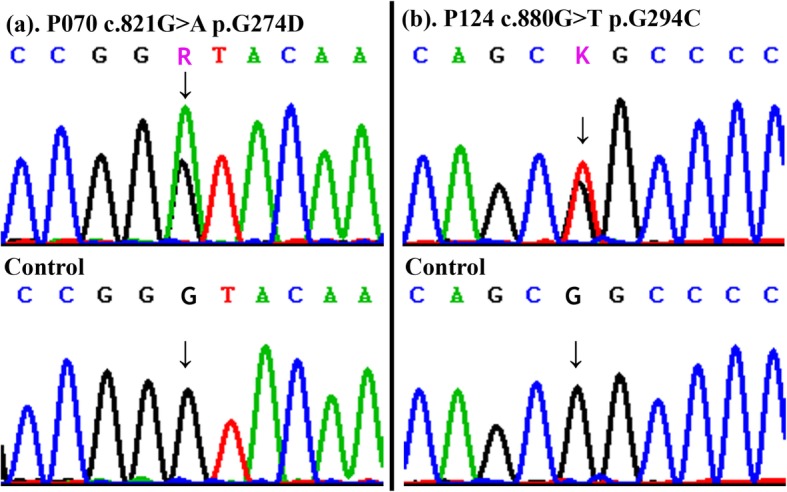
Sequencing chromatograms of the two heterozygous mutants. Panel (**a**) shows the chromatograms of the p.G274D mutants. Panel (**b**) shows the chromatograms of the p.G294C mutants. (“↓” shows mutation sites)

**Fig. 2:**
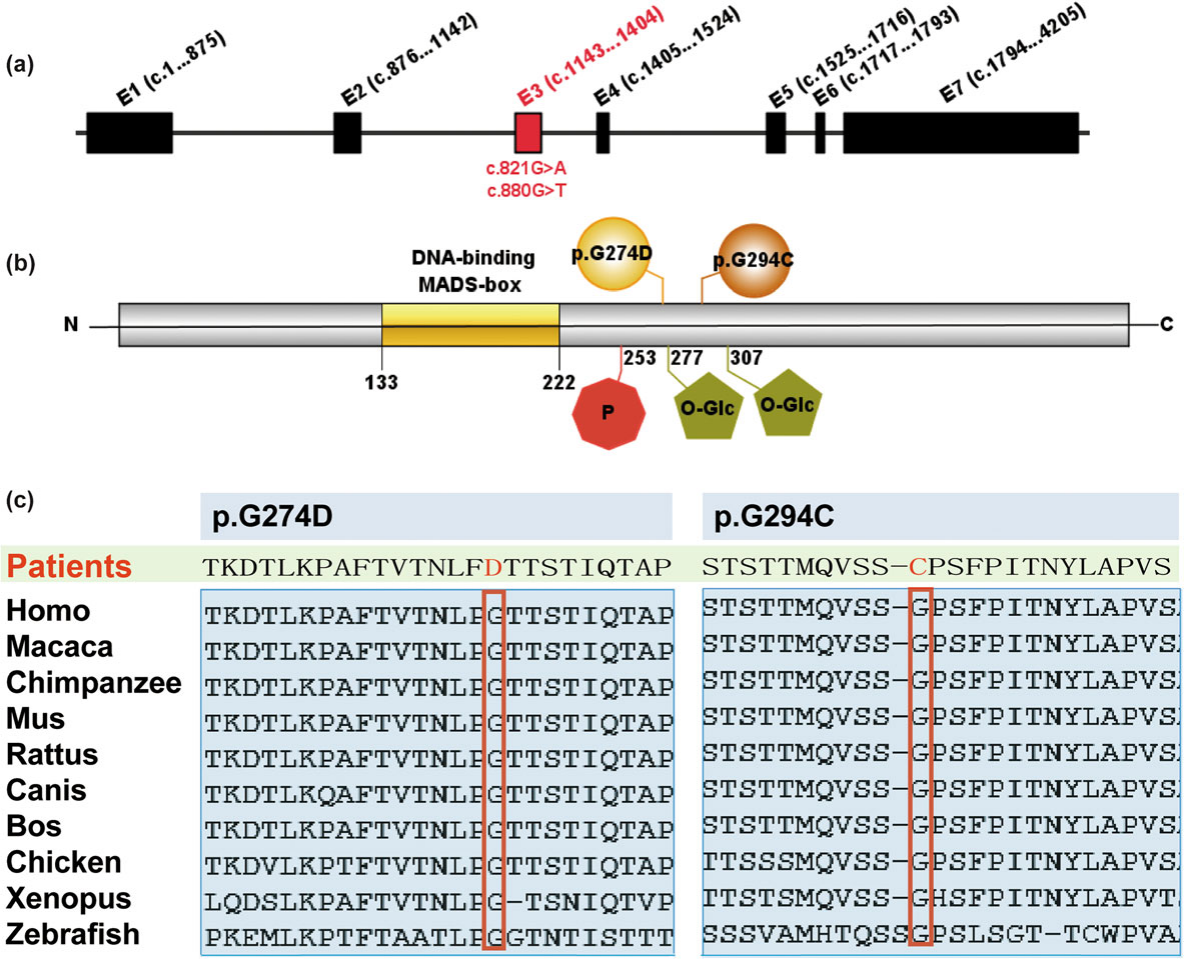
Schematic representation of *SRF* gene and protein. *SRF* gene is 10.212 kb and contains seven exons. **a** and **b** Diagram shows the nucleotide variants (**a**) and amino acid mutations (**b**) of SRF identified in our study cohort. (red octagon containing “p” stands for phosphorylation site; green pentagon stands for O-Glycosylation sites). **c**. Alignment of SRF amino acid residues among different species indicating the level of conservation

We screened 527 sporadic CTD patients (Table 1) for variants of SRF by targeted sequencing. Two nonsynonymous variants were detected: p.G274D in a PA/VSD patient and p.G294C in a TOF/RAA (TOF with right aortic arch) patient, both variants were verified by Sanger sequencing (Fig. 1). The mutations were located in exon 3 (Fig. 2a.), p.G274D and p.G294C were adjacent to the phosphorylation and glycosylation sites of the SRF protein (Fig. 2b.), and the nonpolar hydrophobic glycine (G) residue was substituted with an acidic aspartic acid (D) residue or a polar cysteine (C) residue, respectively. The two mutations were not found in our control group. The allelic frequencies of the two mutants were not found in the Exome Aggregation Consortium (ExAC).

The two variants found in this study result in amino acid substitutions, and the mutation sites are adjacent to the C-terminal phosphorylation site of SRF [30](Fig. 2). Multiple SRF protein sequence alignments showed that the amino acids at position 274 and 294 are highly conserved in vertebrates (Fig. 2c). Bioinformatics analysis suggested that both p.G274D and p.G294C were harmful (Table 3). The conservation and bioinformatics predictions suggested the missense mutants p.G274D and p.G294C may impair SRF function.

### Mutations impair SRF function

**Fig. 3:**
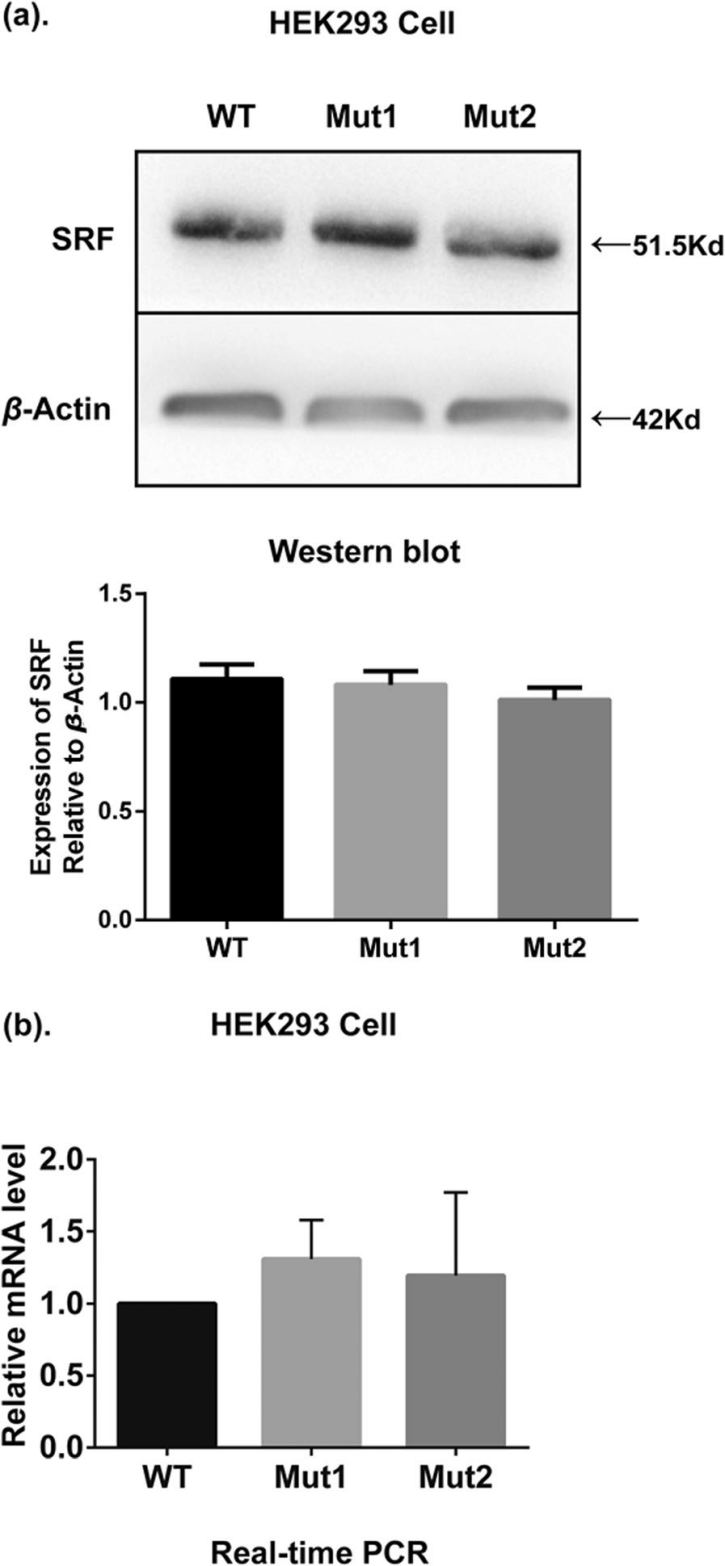
Western blot (**a**) and RT-PCR (**b**) showed there were no obvious differences in protein and gene expressions between mutant and wild-type SRF in HEK293 cells. Anti-SRF antibody and anti-actin antibody (internal control) were used as the primary antibodies. (WT: wild-type, Mut1: p.G274D; Mut2: p.G294C; Blank: pcDNA3.1(+) vector)

To assess whether the mutations affected the expression of SRF, wild-type SRF, SRF-G274D and SRF-G294C vectors were separately transfected into HEK293 cells. qRT-PCR and Western blotting showed that there were no obvious differences in the mRNA and protein expression between the mutant and wild-type SRF (Fig. 3a and b).

**Fig. 4:**
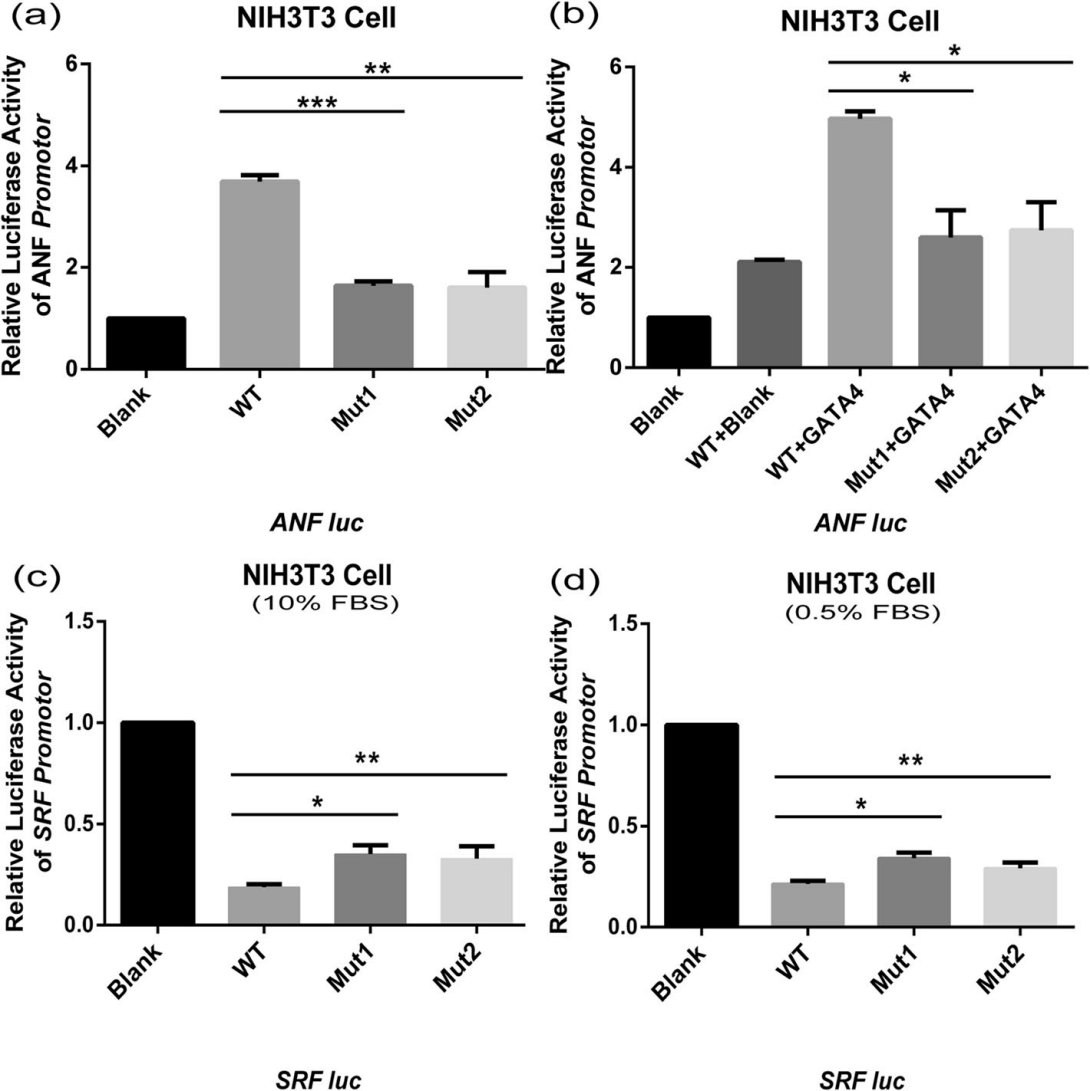
Co-transfected and luciferase assay in NIH3T3, Luciferase activity was used to measure transcription. **a,b**. When transfected alone or co-transfected with GATA4, both Mut1 and Mut2 suggested a decrease in transcriptional activation of ANF promoter when compared to wild-type SRF. (t test, *p < 0.05. **c**. WT vs Mut1: *p* = 0.0002, WT vs Mut2: *p* = 0.003, WT + GATA4 vs Mut1 + GATA4: *p* = 0.0139, WT + GATA4 vs Mut2 + GATA4: *p* = 0.0183). **c**. When NIH3T3 cells were supplemented with 10%FBS, wild-type SRF inhibited the activation of SRF promoter more significantly. (t test, *p < 0.05. WT vs Mut1: *p* = 0.0035, WT vs Mut2: *p* = 0.0343). **d**. When NIH3T3 cells were supplemented with 0.5%FBS, wild-type SRF inhibited the activation of SRF promoter more significantly. (t test, *p < 0.05. WT vs Mut1: *p* = 0.0018, WT vs Mut2: *p* = 0.0289). (WT: wild-type, Mut1: p.G274D; Mut2: p.G294C; Blank: pcDNA3.1(+) vector)

SRF could activate the ANF promoter alone or while interacting with GATA4 [31]. To investigate whether mutant SRF affects ANF activation, we cotransfected the wild-type or mutant SRF plasmid and the ANF promoter plasmid into NIH3T3 cells. At the same time, we also cotransfected the ANF promoter, wild-type or mutant SRF plasmid and GATA4 plasmid into NIH3T3 cells to test whether mutant SRF affected the synergistic effect of SRF and GATA4 on ANF activation (Fig. 4a and b). NIH3T3 cells were starved for 48 h and then harvested to detect the luciferase activity by using a Dual-Glo luciferase assay system. The results showed that wild-type SRF could activate the ANF promoter without GATA4, and the activation was more significant when SRF acted synergistically with GATA4, as expected. However, compared to the activity of wild-type, the transcriptional activity of SRF-G274D and SRF-G294C mutants, alone or in combination with GATA4, at the *ANF* promoter were both receded, and the decrease was more obvious in the G274D mutant (*p* = 0.0002) than in the G294C mutant.

SRF expression could be induced in starved cells with serum stimulating, and previous research showed that there were two CArG box elements located within the first 60 nucleotides upstream of the SRF transcriptional initiation sites and that the transcriptional activity was regulated by SRF binding to the SRE (serum response element) [32]. In addition, transfection studies showed that when NIH3T3 cells were starved for 36 h after transfection and then stimulated with fetal calf serum, the transcription of the luciferase reporter gene *c-fos/*SRE was increased; however, when NIH3T3 cells were kept in 10% FBS medium before and after transfection, the SRF/SRE interaction repressed the *c-fos* reporter activity [30]. To test whether the two SRF mutants impaired the SRF/SRE regulation of the SRF promoter, we cotransfected a *SRFluc* reporter and a wild-type or mutant SRF expression vector into NIH3T3 cells (Fig. 4c and d), and the luciferase activity was measured when NIH3T3 cells were kept in medium supplemented with 10% FBS or 0.5% FBS after transfection. The luciferase activity indicated that SRF inhibited its own activation in both culture conditions. We hypothesize that these effects can be attributed to the negative feedback effect of SRF, resulting in the self-regulation of its expression. However, the repression was significantly reduced in the G274D and G294C mutants compared to wild-type SRF, indicating that these mutants may affect the ability of SRF to inhibit the *SRF/SRE* interaction. Together, our study results suggested that the overexpression of mutant G274D and G294C impaired the transcriptional activity of the ANF promoter and the SRF promoter.

## Discussion

CTDs are complex congenital heart defects that occur during heart development, and lead to a high risk of mortality in perinatal period. The development of cardiac outflow tracts is a complicated process involving major embryological structures including the secondary heart field (SHF) and cardiac neural crest (CNC), and any abnormalities in genetic and environmental factors may disturb the proliferation, differentiation, and migration of the SHF and CNC cells, resulting in conotruncal heart malformations [33–35]. Studies focused on the genetic etiology of CTDs have provided a deeper understanding of CTDs. Many transcription factors and certain chromosomal abnormities related to cardiac development, such as *TBX1*, *GATA4* and *NKx2.5,* have been shown to be involved in the pathogenesis of CTDs [36–38].

SRF is a ubiquitously expressed transcription factor and is involved in cell proliferation and differentiation during embryonic development, especially in the transcriptional activation of many muscle-specific genes [39]. In our study, we investigated the link between SRF mutations and CTDs. We identified novel heterozygous mutations in two of 527 CTD patients (0.3%) who were diagnosed with PA/VSD or TOF/RAA. These two mutations were not found in the 300 control subjects. The two mutations p.G274D and p.G294C, caused amino acid substitutions, and the nonpolar hydrophobic glycine (G) residue was replaced with an acidic aspartic acid (D) residue or a cysteine (C) residue. Both residues are highly conserved in vertebrates based on multiple protein alignment. Analysis with bioinformatics software (Mutation Taster, Polyphen-2 and SIFT) showed that these mutations may be disease-causing (Table 3). The expression of SRF mRNA and protein were not obviously different between the wild-type and mutant proteins. Nevertheless, luciferase assays showed that these two mutations impaired the transcriptional activity of the SRF protein and reduced the ability of SRF to synergize with GATA4 (Fig. 4a and b).

*SRF* is located in 6p21.1, includes eight exons and contains a highly conserved DNA-binding domain called the MADS-box. In mice, the overexpression of mutant SRF resulted in hypertrophic cardiomyopathy in the postnatal heart and increased the expression of fetal cardiac genes [40]. Moreover, the targeted deletion of *SRF* in the developing heart leads to serious myocardial developmental defects, with the reduced expression of several heart-specific genes in mice [41, 42]. In another study using both overexpression and knockdown approaches, SRF was found to be necessary for the induction of *c-fos*, ANF, brain natriuretic peptide, *NCX1*, *α*-actin, and *β*MHC [43]. These SRF-dependent genes are important for the structure or function of the heart. The *α*-actin and *β*MHC genes encode contractile proteins [44, 45], and NCX1 is important for cardiac function through the regulation of Na^+^/Ca^2+^ exchange [46]. ANF is expressed in the myocardial layer at first and is limited to the atrial chamber during heart development [47]. Previous studies suggested that the presence of two SRF binding sites and binding affinity were required for the efficient expression of ANF. In our luciferase assays, the SRF G274D and SRF G294C mutants showed significantly decreased transcriptional activation of the ANF promoter, indicating that these mutations may impair the binding affinity between the mutant proteins and the SRE on the ANF promoter.

GATA4 is an important regulator of cardiogenesis. In the early stage of cardiac development, GATA4-deficient mice showed decreased myocardial proliferation, a lack of mesenchymal cells in the cardiac cushion, and right ventricular dysplasia and GATA4 deletion in the late stage showed double right ventricular outflow with myocardial thinning [48]. SRF interacts with GATA4 and Nkx2.5 and synergistically directs early cardiac gene expression, including the expression of cardiac α-actin (*α*CA), *NCX1*, and *ANF* [49]. The precise cardiac reprogramming effect of *TBX5*, *GATA4*, and *MEF2C* could be enhanced with the addition of *MYOCD* and *SRF* [50]. SRF acts as an anchoring protein to recruit GATA4 and NKx2.5 to generate transcriptional complexes that promote the efficient expression of αCA; additionally, the recruitment of GATA4 and NKx2.5 by SRF strongly enhances the SRF DNA-binding affinity [51, 52], and the early depletion of SRF in the E9.5 hearts of transgenic mice results in the downregulation of GATA4, myocardin and NKx2.5. Our luciferase assay results show that the two mutants (p.G274D and p.G294C) found in our cohort significantly reduced the synergistic effect of SRF and GATA4 on the activation of the ANF promoter (Fig. 4b). GATA4 deletion in the late stage of heart development results in DORV with myocardial thinning in mice [48]. We speculate that these two mutants impair the synergy of SRF with GATA4 and were involved in the pathogenesis of CTDs in these two patients.

Studies have shown that the C-terminal phosphorylation of SRF, which is confined to amino acids 206–289, mainly contributes to transcriptional activation or repression [30]. When NIH3T3 cells were stimulated for 36 h with 10% FBS after transfection, the SRE/SRF interaction repressed the transcriptional activity of the *c-fos* promoter which contains an SRE [30, 53]. In accordance with the view of Janknecht Ralf that the C-terminal phosphorylation of SRF was confined to amino acids 206–289, in our study, the p.G274D and p.G294C mutations were adjacent to the C-terminal phosphorylation sites of SRF. To examine the self-regulation of SRF transcriptional activity, we cotransfected the SRF promoter and the wild-type or mutant SRF plasmid into NIH3T3 cells and cultured the cells in 10% FBS medium or 0.5% FBS medium for another 48 h after transfection. Then we performed a luciferase assay. The results showed that SRF inhibits its own expression regardless of the presence or absence of serum, and mutant SRF presented less obvious inhibitory effects than wild-type SRF. We speculated that SRF has a negative feedback effect on its regulation.

The novel p.G274D and p.G294C SRF mutations were detected in two patients in our cohort, who were diagnosed with PA/VSD or TOF/RAA. Bioinformatics analysis suggested that both mutations were disease-causing. Although changes in the mRNA and protein levels were not obvious, the function of the mutant protein may be impaired. By verifying the reduction in the transcriptional activation or repression of mutant SRF, we hypothesize that SRF gene mutations contribute to the risk of CTDs.

## Conclusion

These two novel mutations found in our studing cohort may be pathogenic. The transcriptional activity of the mutant proteins decreased, affecting the expression of downstream genes, which may be one of the causes of heart defects in the two CTD patients. Our findings may provide a genetic reference for the pathogenesis of CTDs.

## Data Availability

The gene or protein sequence analysed during the current study are available in the Gene database (https://www.ncbi.nlm.nih.gov/gene/) and the Protein (https://www.ncbi.nlm.nih.gov/home/protein/) databases of National Center of Biotechnology Information (NCBI) repository, the relevant accession numbers of all SRF gene (NM_003131.4 and NC_000006.12) and SRF protein sequences (NP_003122.1, XP_001093365, XP_518487, NP_065239.1, NP_001102772, XP_852302.1, NP_001192945, NP_001239070, XP_002942523, NP_001103996) have been noted at the corresponding positions in the Methods section. The MutationTaster (http://www.mutationtaster.org/), SIFT (http://sift.jcvc.org/www/SIFT_enst_submit.html) and Polyphen-2 (http://genetics.bwh.havard.edu/pph2/) were used to predict the bioinformatics of mutant SRF protein.

## Abbreviations

CTD: Conotruncal heart defects
TOF: Tetralogy of Fallot
DORV: Double outlet right ventricle
TGA: Transposition of the great arteries
TA: Truncus arteriosus
IAA: Interrupted aortic arch
PA/VSD: Pulmonary atresia/ventricular septal defect
SRF: Serum response factor
SHF: Secondary heart field
CNC: Cardiac neural crest
SRE: Serum response element
